# Outcomes after radioscapholunate arthrodesis for intra-articular malunion of distal radius fractures

**DOI:** 10.1007/s00590-024-03934-6

**Published:** 2024-04-23

**Authors:** Yasser Safoury, Ahmed Afifi, Ahmed Farghaly, Omar Khalid

**Affiliations:** Hand, Upper Limb, and Microsurgery Unit, Department of Orthopaedic Surgery, Faculty of Medicine, Cairo University, Cairo, Egypt

**Keywords:** Radioscapholunate, Arthrodesis, Malunited distal radius, Distal scaphoid excision, Midcarpal osteoarthritis

## Abstract

**Purpose:**

To study the clinical, radiological, and functional outcomes after of radioscapholunate (RSL) fusion for intra-articular malunion of the distal radius.

**Methods:**

This retrospective study included 26 patients (17 males and 9 females) with intra-articular malunion of distal radius fractures who underwent RSL arthrodesis using locked miniplates (without distal scaphoid excision) between 2012 and 2020. Their mean age was 43 years (range, 32–56). Patients were assessed radiographically for union and clinically for range of motion, grip strength, and pain (assessed by Visual Analogue Scale (VAS) for pain). Functional evaluation was performed by using the Mayo modified wrist score (MMWS) and the Disabilities for the Arm, Shoulder, and Hand (DASH) questionnaire.

**Results:**

All patients showed complete healing at the fusion site after a mean of 8.7 weeks (range, 8–12). The mean follow-up period was 72 months (range, 60–84). The pinch strength improved from a mean of 6.2 kg (range, 3–12) to a mean of 9.8 kg (range, 5–18) which represents 80% of the contralateral side. The mean pinch strength was 7 kg (range, 5–18) which presents 80% of the other side. VAS for pain showed a mean improvement of 72.6%. The DASH score improved to a mean of 19.2 (range, 14–24). The MMWS improved to a mean of 68 (range, 45–86). At the final follow-up period, no degenerative changes were detected in the midcarpal joint.

**Conclusion:**

RSL arthrodesis (using locked miniplates without distal scaphoid excision) is a reliable surgical procedure to manage cases of radiocarpal OA after intra-articular malunion of distal radius fractures with good clinical and radiological outcomes.

**Level of evidence:**

Level IV- therapeutic.

## Introduction

Radioscapholunate (RSL) fusion is a well-known salvage procedure to treat radiocarpal osteoarthritis with an intact midcarpal joint. This type of limited wrist fusion has proved to be a reliable and safe procedure to improve pain and maintain some wrist motion in patients that had signs of posttraumatic osteoarthritic changes limited to the radiocarpal articulation [1].

There are several techniques for RSL fusion described in the literature with different surgical approaches (dorsal or volar) and methods of fixation (K-wires, staples, compression screws, or locked angled plates). Also, there is no consensus about the value of distal scaphoid excision (DSE) or even adding a triquetrum excision. Moreover, the need for bone grafting is still debatable (iliac crest, distal radius, or from the excised bone). Results in the previous studies were evaluated according to the range of motion, the rate of union, and the development of late complications as osteoarthritis (OA) of the midcarpal joint. Comparing these results in the literature is difficult as there are a lot of variable factors included in each study [1–6].

The previous reports about the outcomes of RSL fusion were conducted on heterogeneous groups of patients. The aim of this study was to assess the outcomes of RSL fusion using locked miniplates (done for patients with intra-articular malunion of distal radius fractures) clinically and radiologically after an average of 6 years.

## Materials and methods

### Study design and setting

The Institutional Review Board (IRB) approved a single-center, retrospective study including all patients treated with RSL fusion who were eligible for enrollment. The study was conducted between 2012 and 2020 at an academic Level 1 Trauma Center.

### Participants

**Fig. 1:**
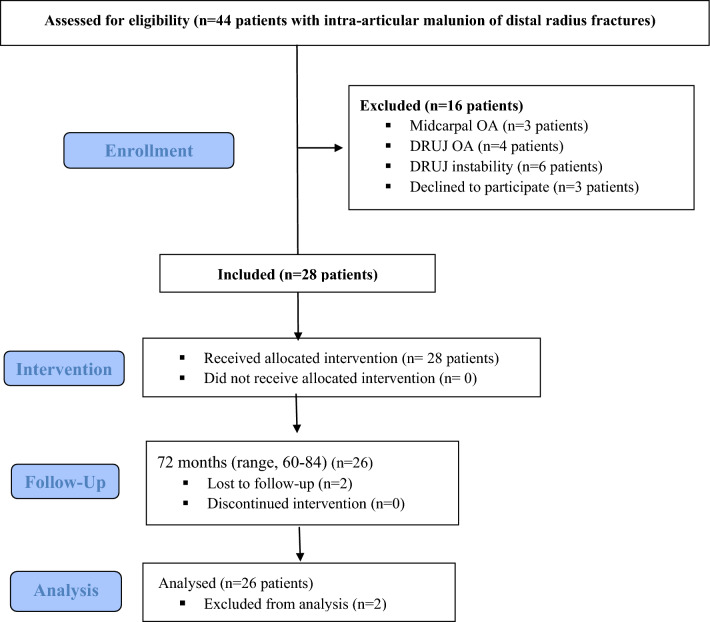
Flowchart of the participants

This study included 26 patients (17 males and 9 females) with intra-articular malunion of distal radius fractures who underwent RSL arthrodesis. Their mean age was 43 years (range, 32–56 years). The indications for surgery were pain and limited activity after a neglected intra-articular fracture of the distal radius or failure after surgical reduction and fixation of the primary fracture. Patients with associated midcarpal OA on computed tomography (CT) scans and patients with distal radioulnar joint (DRUJ) OA or instability were excluded (Fig. 1). Patients were presented to our service after a mean of 10.7 months from the initial injury (range, 6–18 months) (Table 1). Preoperative X-rays (posteroanterior and lateral views) and CT scans were done to identify the degree of OA in the radiocarpal (RC) joint and exclude any midcarpal joint involvement.

**Table 1:** Patients' demographics

Mean age	43 years (range, 32–56)
Male: female	17 (65.4%): 9 (34.6%)
Dominant: non-dominant hands	18 (69.2%): 8 (30.8%)
Occupation (manual worker/office worker/housewife)	2/19/5
Neglected fractures	20 (76.9%)
Failed fixation	6 (23.1%)
Interval from injury to surgery	6 months (range, 3–18)

### Surgical technique

**Fig. 2:**
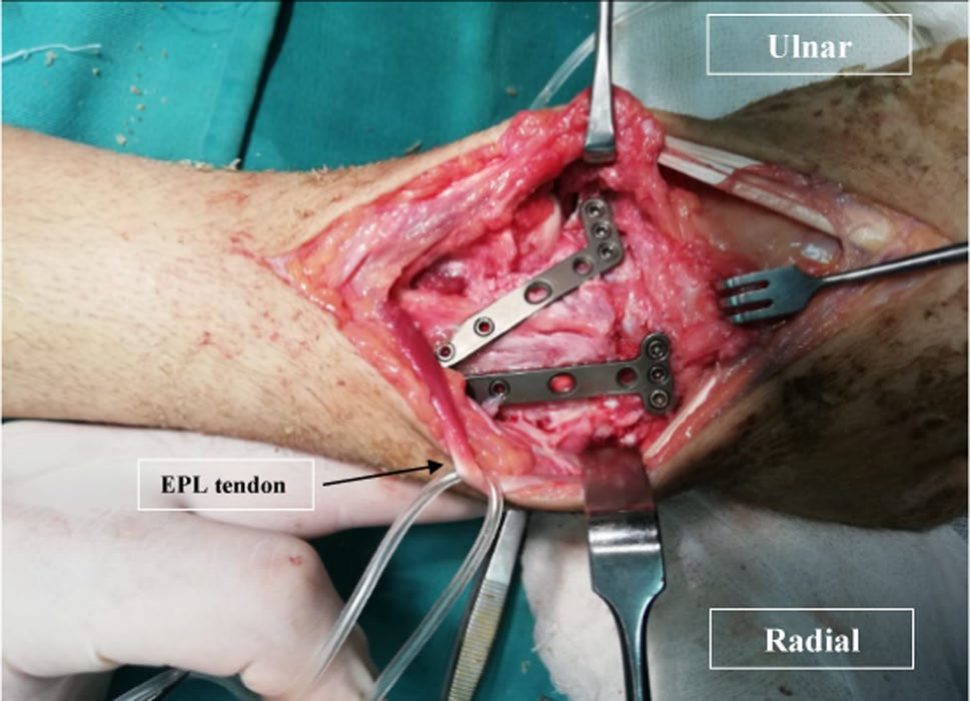
The surgical procedure showing no opening of the midcarpal joint and fixation with 2 miniplates separately for both the scaphoid and the lunate. *EPL* Extensor Pollicis Longus

All the surgical procedures were performed by the senior author (YS). Under general anesthesia and a tourniquet control, a midline dorsal skin incision was made. The dorsal sensory branches of the radial nerve were identified and protected. Then, the extensor retinaculum was incised with an oblique incision along with the third compartment. The extensor pollicis longus (EPL) tendon was freed out of the fibro-osseous groove and retracted with a tape. Partial wrist denervation was performed for all patients (posterior interosseous nerve neurectomy). A limited ligament preserving capsulotomy was done to expose the articular surfaces of the radius and the carpal bones only without exposing the midcarpal joint. The distal radius and the reciprocal surfaces of the scaphoid and the lunate were decorticated to bleeding cancellous bone. It was important at this stage to ensure that the articular margins of the scaphoid and the lunate were anatomically reduced and aligned with the distal radius. Slight distraction was done to restore the carpal height and prevent ulnar impaction. Iliac crest bone graft was used to fill the gap at the fusion site. Fixation was then done keeping the achieved position by two locked 2-mm miniplates (Monoloc 2-mm locking plates, Medtronic) separately for both the scaphoid and the lunate (Fig. 2).

The dorsal capsular retinacular flaps were then closed as one layer leaving the EPL tendon out of the extensor retinaculum. A plaster slab was applied for a period of 4 weeks encouraging full hand motion. Then, patients used removable thermoplastic wrist splints and were followed up by plain x-rays until full union.

### Outcome evaluation

All patients were subjected to a strict postoperative protocol at 2, 6, and 8 weeks then at 3, 6, 12, 18, and 24 months after surgery then every year till the final follow-up. The primary outcome measure was union of arthrodesis. Successful fusion was defined as the crossing of clear bony trabeculae from the radius to the carpus on plain posteroanterior and lateral wrist radiographs.

Secondary outcome measures included the wrist range of motion (measured by a goniometer), grip strength (using a Jamar dynamometer), pinch strength (using a Jamar pinch gauge), and the Visual Analogue Scale (VAS) for pain. VAS was calculated by using a ruler to measure the distance (mm) between the “no pain” mark and the patient's mark on a 10-cm line, yielding a range of scores from 0 to 100. Pain intensity was classified as: no pain (0–4 mm), mild pain (5–44 mm), moderate pain (45–74 mm), and severe pain (75– 100 mm) [7, 8]. Functional evaluation was carried out using the modified Mayo wrist score (MMWS) [9] and the disabilities of the arm, shoulder, and hand (DASH) score [10].

### Statistical methods

Data were summarized using mean, standard deviation, median, minimum and maximum in quantitative data and using frequency (count) and relative frequency (percentage) for categorical data. The Shapiro test was used for proving a normal distribution of the numerical variables. For comparison of serial measurements within each patient, the nonparametric Wilcoxon signed-rank test was used. An exact test was used instead when the expected frequency is less than 5. *P*-values less than 0.05 were considered statistically significant.

## Results

All patients showed complete healing at the fusion site after a mean of 8.7 (range, 8–12) weeks. At the final follow-up period, no degenerative changes (joint narrowing, subcortical sclerosis, or osteophyte formation) were detected at the STT or the midcarpal joints. The mean follow-up period was 72 (range, 60–84) months.

None of the patients had pain in the distal radioulnar joint or forearm rotation. Therefore, no additional procedures were required at the distal radioulnar joint during the follow-up period.

The grip strength was improved from a mean of 15.3 kg (range, 8–30) to a mean of 24.7 kg (range, 12–42) which represents 70% of the contralateral side. The pinch strength improved from a mean of 6.2 kg (range, 3–12) to a mean of 9.8 kg (range, 5–18) which represents 80% of the contralateral side. VAS improved from a mean of 62.7 (range, 40–90) to a mean of 16.1 (range, 10–20). The Mayo modified wrist score improved from a mean of 32.1 (range, 10–55) to a mean of 68.7 (range, 45–85). The DASH score improved from a mean of 65.9 (range, 40–82) to a mean of 19.2 (range, 14–24). Table 2 shows the changes in the wrist range of motion and the other outcomes.

**Table 2:** Comparison of the outcome measures before surgery and at the final follow-up period

	Preoperative	At the final follow-up	*P*-value
Range of motion			
Flexion	44.4° (range, 30–50)	28.4° (range, 20–35)	< 0.001
Extension	55.9° (range, 48–72)	40.2° (range, 33–45)	< 0.001
Radial deviation	18.5° (range, 12–22)	9° (range, 7–12)	< 0.001
Ulnar deviation	28.7° (range, 20–32)	19.9° (range, 15–24)	< 0.001
Grip strength	15.3 (range, 8–30)	24.7 (range, 12–42)	< 0.001
Pinch strength	6.2 (range, 3–12)	9.8 (range, 5–18)	< 0.001
VAS	62.7 (range, 40–90)	16.1 (range, 10–20)	< 0.001
MMWS	32.1 (range, 10–55)	68.7 (range, 45–85)	< 0.001
DASH	65.9 (range, 40–82)	19.2 (range, 14–24)	< 0.001

**Fig. 3:**
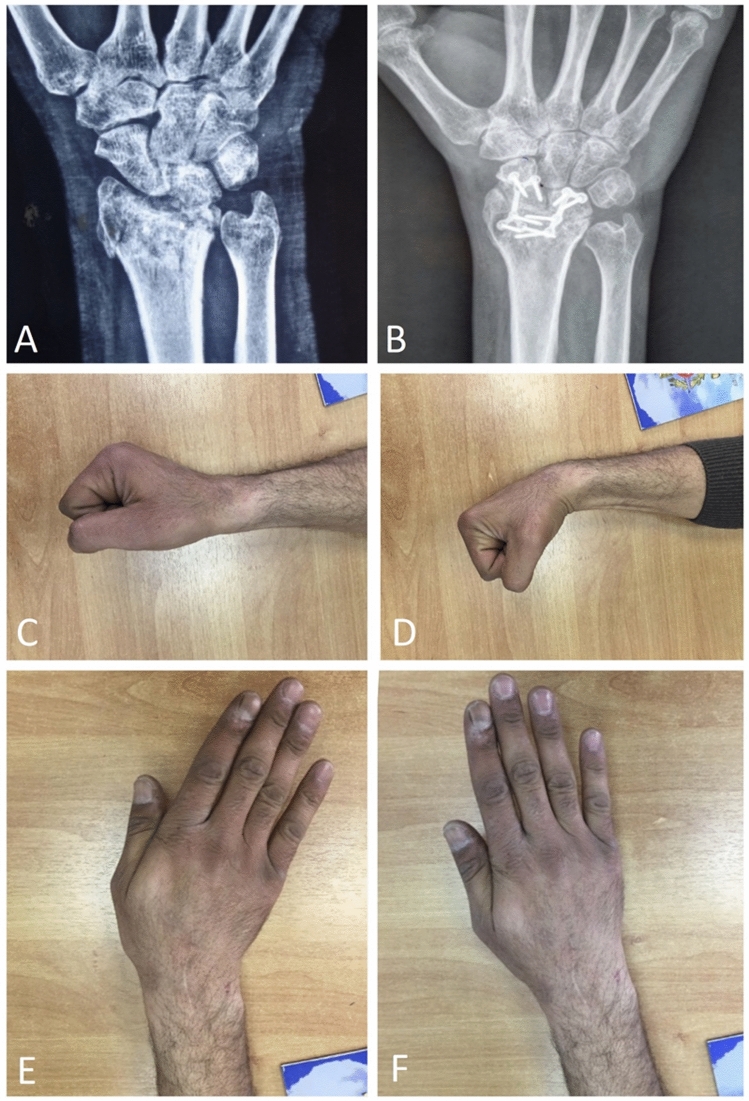
A Case example of RSL fusion after intra-articular distal radius malunion, **A** preoperative posteroanterior wrist radiograph, **B** final posteroanterior radiograph after union of RSL arthrodesis, **C** final wrist extension range, **D** final wrist flexion range, E: final ulnar deviation range, **F** final radial deviation range (limited)

There were few complications as screw irritation in one patient to whom hardware removal was done 6 months after surgery. Three patients developed temporary paresthesia from irritation to the branches of the superficial radial nerve that improved with time. One patient was not satisfied with his result as he was not able to return to his original job as a manual laborer. Figure 3 shows radiographs and clinical photographs of a case example.

## Discussion

The non-union rate after RSL fusion in the published studies is a major concern. It has ranged from 6 to 27% which could be due to the lack of rigid bone fixation, especially when using K-wires. Some studies reported that the non-union rate was high in patients to whom DSE was not done. However, many other unevaluated factors could explain the incidence of non-union, such as the number of surgeons who were involved, the different surgical skills, the type of the implants used for fixation, and other factors, such as smoking or previous steroid therapy [1, 5, 11–16]. In this series, there were no non-unions possibly due to the rigid fixation of each bone separately with locked plates and the addition of corticocancellous iliac crest bone graft. The use of low-profile locked miniplates also abolished the disadvantages of the other fixation methods, such as KW (no compression), non-locked miniplates (dorsal impingement or loss of fixation) [1].

The results of this concerning the range of motion are consistent with the studies that examine the wrist motion from a functional standpoint (the dart-throwing motion) [17]. Brumfield and Champoux demonstrated that the wrist accomplishes most activities between 10° flexion and 35° extension. Palmar et al., using more sophisticated measuring techniques, demonstrated that the functional wrist range of motion for most daily activities was 5° flexion, 30° extension, 10° radial deviation, and 15° ulnar deviation [18, 19].

Several studies have suggested DSE during this procedure to allow a greater range of motion especially radial deviation and also to have a higher rate of union or even to decrease the possibility of OA of the scaphotrapeziotrapezoid (STT) joint [1, 11, 20–22]. Other studies showed that although DSE may improve short-term range of motion, but it comes at the cost of increasing the contact pressure in the remaining midcarpal joint (mainly the lunocapitate joint) that further increases the incidence of midcarpal arthritis [23–25].

Mühldorfer-Fodor et al. [11] retrospectively compared the outcomes of RSL arthrodesis alone and with a distal scaphoidectomy. They concluded that the results were comparable in both groups regarding pain relief and functional improvement. The rates of midcarpal degeneration were similar in both groups with no long-term radiographic follow-up.

The absence of osteoarthritis and ulnar abutment in this series could be explained by the proper alignment, the restoration of the carpal height, and not performing DSE that increases the stresses on the midcarpal joint. This is supported by other studies by Shin et al. and Galvis et al. [25, 26].

Quadlbauer et al. [12] reported the outcomes of volar RSL arthrodesis and DSE in 14 patients with radiocarpal osteoarthritis secondary to malunited distal radius. Union was achieved in all patients without the development of midcarpal OA after a mean follow-up of 63 months. However, this study is limited by being retrospective with a small sample size and the absence of a control group.

This is a large series of patients with a long follow-up, done and followed-up by one group of surgeons. Many of the published studies were conducted by multiple different surgeons with different indications, techniques of fixation, and grafting with variable follow-up periods. Also, the literature review revealed a gap concerning the assessment of midcarpal OA clinically and radiologically and with no clarification of the long-term impact of both excision of the distal pole of the scaphoid and the triquetrum on the midcarpal joint. The main shortcomings of our series are being retrospective, the lack of a control group, and the use of different designs of miniplates. A randomized clinical trial with and without distal scaphoid excision is recommended. Table 3 shows a comparison of our results with other studies.

**Table 3:** Comparison between our study and other studies

	Design	No. of cases	Indications	Approach	Fixation	DSE	Mean FU	Union	VAS	MMWS	DASH	Grip strength	Non-union	Degenerative OA
Nagy and Büchler [5]	Retrospective	15	Sequelae of DRF*	Dorsal	Plates, staples	No	8 yrs	11 weeks	–	–	–	63%	4 (27%)	7 patients (47%)
Garcia-Elias et al. [1]	Retrospective	16	Different	Dorsal	KW*, T-plate, Herbert screws	Yes	3.1 yrs	–	–	–	–	–	0	2 midcarpal OA (13%)
Mühldorfer-Fodor et al. [11]	Retrospective	Group A: 20 Group B: 15	Painful posttraumatic radiocarpal osteoarthritis	Dorsal	KW	Group A: 20 (yes) Group B: 15 (no)	2.3 yrs	–	–	–	–	–	3 in group B	3 patients
Quadlbauer et al. [12]	Retrospective	11	Intra-articular malunion incurred after a surgically treated DRF	Volar	Plates	Yes	5.3 yrs	All	2.2	–	27	28 kg (80% of the sound side)	0	No midcarpal OA All patients had asymptomatic DRUJ OA
Montoya-Faivre et al. [16]	Retrospective	19	Different	Dorsal	Different methods	Yes/No	4.4 yrs	–	3	57.2	44.5 (QuickDASH)	71%	4 (21%)	7 midcarpal OA (37%)
Ha et al. [14]	Prospective	11	Different	Dorsal		Yes	14.8 yrs	–	–	–	–	–	0	Not reported
Degeorge et al. [13]	Retrospective	75	Painful posttraumatic radiocarpal osteoarthritis	Dorsal	Different methods	33 No 26 Yes 16 DSE + ET	9.1 yrs	–	–	–	–	–	4 (9%)	14 (33%)
Our series 2021	Prospective	26	Intra-articular MUDR*	Dorsal	Plates	No	6 yrs	8.7 weeks	16.1	68	19.2	24 kg (70% of the sound side)	0	0

^*^DRF Distal radius fractures, *KW* Kirschner wires, *MUDR* Malunited distal radius

To conclude, RSL arthrodesis using locked miniplates without DSE is a reliable surgical option for intra-articular malunion of the distal radius with good clinical and radiological outcomes.

## Data Availability

Available.
