# Correction to: Outcomes after radioscapholunate arthrodesis for intra‑articular malunion of distal radius fractures

**DOI:** 10.1007/s00590-024-04013-6

**Published:** 2024-07-16

**Authors:** Yasser Safoury, Ahmed Afifi, Ahmed Farghaly, Omar Khalid

**Affiliations:** Hand, Upper Limb, and Microsurgery Unit, Department of Orthopaedic Surgery, Faculty of Medicine, Cairo University, Cairo, Egypt

**Correction to: European Journal of Orthopaedic Surgery & Traumatology** 10.1007/s00590-024-03934-6

In the original version of this article, there are typing mistakes. In the abstract, “Purpose” section which previously read

To study the clinical, radiological, and functional outcomes after of radioscapholunate (RSL) fusion for intraarticular malunion of the distal radius.

Should have read

To study the clinical, radiological, and functional outcomes after radioscapholunate (RSL) fusion for intraarticular malunion of the distal radius.

In the abstract, “Results” section which previously read

All patients showed complete healing at the fusion site after a mean of 8.7 weeks (range, 8–12). The mean followup period was 72 months (range, 60–84). The pinch strength improved from a mean of 6.2 kg (range, 3–12) to a mean of 9.8 kg (range, 5–18) which represents 80% of the contralateral side. The mean pinch strength was 7 kg (range, 5–18) which presents 80% of the other side. VAS for pain showed a mean improvement of 72.6%. The DASH score improved to a mean of 19.2 (range, 14–24). The MMWS improved to a mean of 68 (range, 45–86). At the final follow-up period, no degenerative changes were detected in the midcarpal joint.

Should have read

All patients showed complete healing at the fusion site after a mean of 8.7 weeks (range, 8–12). The mean followup period was 72 months (range, 60–84). Grip strength improved to a mean of 24.7 kg (range, 12–42). Pinch strength improved to a mean of 9.8 kg (range, 5–18). VAS for pain improved to a mean of 16.1 (range, 10–20). The DASH score improved to a mean of 19.2 (range, 14–24). The MMWS improved to a mean of 68.7 (range, 45–85). At the final follow-up period, no degenerative changes were detected in the midcarpal joint.

In the “Introduction” section, second sentence which previously read

This type of limited wrist fusion has proved to be a reliable and safe procedure to improve pain and maintain some wrist motion in patients that had signs of posttraumatic osteoarthritic changes limited to the radiocarpal articulation [1].

Should have read

This type of limited wrist fusion has proved to be a reliable and safe procedure to improve pain and maintain some wrist motion in patients who had signs of posttraumatic osteoarthritic changes limited to the radiocarpal articulation [1].

In the “Outcome evaluation” section, second sentence which previously read

The primary outcome measure was union of arthrodesis. Successful fusion was defined as the crossing of clear bony trabeculae from the radius to the carpus on plain posteroanterior and lateral wrist radiographs.

Should have read

The primary outcome measure was the union of arthrodesis. Successful fusion was defined as the crossing of clear bony trabeculae from the radius to the carpus on plain posteroanterior and lateral wrist radiographs.

In the “Discussion” section, first sentence of the second paragraph which previously read

The results of this concerning the range of motion are consistent with the studies that examine the wrist motion from a functional standpoint (the dart-throwing motion) [17].

Should have read

The results of this study concerning the range of motion are consistent with the studies that examine the wrist motion from a functional standpoint (the dart-throwing motion) [17].

In the “Discussion” section, second sentence of the third paragraph which previously read

Other studies showed that although DSE may improve short-term range of motion, but it comes at the cost of increasing the contact pressure in the remaining midcarpal joint (mainly the lunocapitate joint) that further increases the incidence of midcarpal arthritis [23–25].

Should have read

Other studies showed that although DSE may improve the short-term range of motion, it comes at the cost of increasing the contact pressure in the remaining midcarpal joint (mainly the lunocapitate joint) that further increases the incidence of midcarpal arthritis [23–25].

In the “Discussion” section, first sentence of the seventh paragraph which previously read

This is a large series of patients with a long follow-up, done and followed-up by one group of surgeons.

Should have read

This is a large series of patients with a long follow-up, done and followed up by one group of surgeons.

In the last row of the Table 3, first column should be “Our series” instead of “Our series 2021” and second column should be “Retrospective” instead of “Prospective” and eleventh column should be “68.7” instead of “68” and thirteenth column should be “24.7 kg (70% of the sound side)” instead of “24 kg (70% of the sound side)”. The correct Table 3 should have been.

**Table 3:** Comparison between our study and other studies

	Design	No. of cases	Indications	Approach	Fixation	DSE	Mean FU	Union	VAS	MMWS	DASH	Grip strength	Non-union	Degenerative OA
Nagy and Büchler [5]	Retrospective	15	Sequelae of DRF*	Dorsal	Plates, staples	No	8 yrs	11 weeks	–	–	–	63%	4 (27%)	7 patients (47%)
Garcia-Elias et al. [1]	Retrospective	16	Different	Dorsal	KW*, T-plate, Herbert screws	Yes	3.1 yrs	–	–	–	–	–	0	2 midcarpal OA (13%)
Mühldorfer-Fodor et al. [11]	Retrospective	Group A: 20 Group B: 15	Painful posttraumatic radiocarpal osteoarthritis	Dorsal	KW	Group A: 20 (yes) Group B: 15 (no)	2.3 yrs	–	–	–	–	–	3 in group B	3 patients
Quadlbauer et al. [12]	Retrospective	11	Intra-articular malunion incurred after a surgically treated DRF	Volar	Plates	Yes	5.3 yrs	All	2.2	–	27	28 kg (80% of the sound side)	0	No midcarpal OA All patients had asymptomatic DRUJ OA
Montoya-Faivre et al. [16]	Retrospective	19	Different	Dorsal	Different methods	Yes/No	4.4 yrs	–	3	57.2	44.5 (QuickDASH)	71%	4 (21%)	7 midcarpal OA (37%)
Ha et al. [14]	Prospective	11	Different	Dorsal		Yes	14.8 yrs	–	–	–	–	–	0	Not reported
Degeorge et al. [13]	Retrospective	75	Painful posttraumatic radiocarpal osteoarthritis	Dorsal	Different methods	33 No 26 Yes 16 DSE + ET	9.1 yrs	–	–	–	–	–	4 (9%)	14 (33%)
Our series	Retrospective	26	Intra-articular MUDR*	Dorsal	Plates	No	6 yrs	8.7 weeks	16.1	68.7	19.2	24.7 kg (70% of the sound side)	0	0

*DRF Distal radius fractures, *KW* kirschner wires, *MUDR* Malunited distal radius

